# Different Roles of *N*-Terminal and *C*-Terminal Domains in Calmodulin for Activation of *Bacillus anthracis* Edema Factor

**DOI:** 10.3390/toxins7072598

**Published:** 2015-07-13

**Authors:** Carolin Lübker, Stefan Dove, Wei-Jen Tang, Ramona J. Bieber Urbauer, Jackob Moskovitz, Jeffrey L. Urbauer, Roland Seifert

**Affiliations:** 1 Institute of Pharmacology, Hannover Medical School, Carl-Neuberg-Str. 1, Hannover D-30625, Germany; E-Mail: luebker.carolin@mh-hannover.de; 2 Institute of Pharmacy, University of Regensburg, Universitätsstr. 31, Regensburg D-93053, Germany; E-Mail: stefan.dove@chemie.uni-regensburg.de; 3 Ben May Department of Cancer Research, University of Chicago, 929 E. 57th Street, Chicago, IL 60637, USA; E-Mail: wtang@bsd.uchicago.edu; 4 Department of Chemistry, University of Georgia, 140 Cedar Street, Athens, GA 30602-2556, USA; E-Mails: ramonau@uga.edu (R.J.B.U.); urbauer@uga.edu (J.L.U.); 5 Department of Pharmacology and Toxicology, University of Kansas, 1251 Wescoe Hall Drive, Lawrence, KS 66045, USA; E-Mail: moskovij@ku.edu

**Keywords:** *Bacillus anthracis* edema factor, adenylyl cyclase toxin, (oxidized) calmodulin, (oxidized) calmodulin mutants

## Abstract

*Bacillus anthracis* adenylyl cyclase toxin edema factor (EF) is one component of the anthrax toxin and is essential for establishing anthrax disease. EF activation by the eukaryotic Ca^2+^-sensor calmodulin (CaM) leads to massive cAMP production resulting in edema. cAMP also inhibits the nicotinamide adenine dinucleotide phosphate (NADPH)-oxidase, thus reducing production of reactive oxygen species (ROS) used for host defense in activated neutrophils and thereby facilitating bacterial growth. Methionine (Met) residues in CaM, important for interactions between CaM and its binding partners, can be oxidized by ROS. We investigated the impact of site-specific oxidation of Met in CaM on EF activation using thirteen CaM-mutants (CaM-mut) with Met to leucine (Leu) substitutions. EF activation shows high resistance to oxidative modifications in CaM. An intact structure in the C-terminal region of oxidized CaM is sufficient for major EF activation despite altered secondary structure in the N-terminal region associated with Met oxidation. The secondary structures of CaM-mut were determined and described in previous studies from our group. Thus, excess cAMP production and the associated impairment of host defence may be afforded even under oxidative conditions in activated neutrophils.

## 1. Introduction

Anthrax disease caused by *Bacillus anthracis* infection continues to be a significant anthropozoonosis in developing countries, despite the successes of veterinary vaccines [1,2,3]. Anthrax is also a threat as a bioterrorism weapon [3,4], and newer problems are deaths from use of illegal injectable drugs like heroin contaminated with anthrax spores [3]. Two component proteins of the anthrax toxin, the adenylyl cyclase (AC), referred to as edema factor (EF), and lethal factor (LF), respectively, penetrate into host cells via interaction with protective antigen (PA) protein [5,6]. PA builds a prepore complex with EF and LF after binding to CM2G2/TEM8 receptors located on host immune cells. The complex internalizes into acid vacuoles, and pH-dependent processes mediate the release of the toxins into host cytosol [5,6]. Because EF is central to the pathology of anthrax by inducing edema and shock [7,8], potent and selective EF inhibitors would be very useful tools for combating anthrax [9]. Currently, the most potent EF inhibitors target the catalytic site, but membrane-permeability and selectivity of compounds are of concern [9].

Another potential target for EF inhibitors is the interaction site of EF with its activator calmodulin (CaM) [5,6,10]. CaM is a eukaryotic Ca^2+^-sensor in host cells including neutrophils, a preferential target cell of EF [11]. The high AC activity of CaM-stimulated EF induces a massive increase of cAMP levels that severely disrupt cellular signalling and homeostasis [12]. The high intracellular cAMP levels also significantly inhibit production of superoxide that is catalyzed by nicotinamide adenine dinucleotide phosphate (NADPH)-oxidase, an important component of the host defense system in activated neutrophils [11]. Thus, EF facilitates bacterial growth and promotes development of anthrax disease by interrupting this important host defense mechanism [3,6].

Superoxide and other reactive oxygen species (ROS) can also damage mammalian proteins by oxidation of susceptible amino acid residues, including methionine (Met) [13,14]. In CaM all nine Met (M36, M51, M71, M72, M76, M109, M124, M144 and M145) can be oxidized *in vivo* and *in vitro* to the (*S*)- and (*R*)-diastereoisomers of methionine sulfoxide (MetSO) [15,16].

Activation of CyaA of *Bordetella pertussis*, a CaM-stimulated AC toxin exhibiting similar catalytic and regulatory mechanisms to EF [10,17], is dramatically impaired by *in vitro* oxidized CaM [18]. All these data prompted us to study activation of EF by *in vitro* oxidized CaM. In order to analyze the impact of the redox state of distinct Met on EF activation, we used 13 CaM-mutants (CaM-mut) with site-specific Met to leucine (Leu) substitutions (Table 1), which were previously analyzed with regard to activation of mammalian membranous adenylyl cyclase 1 (AC1) [19]. Leu is comparable to Met in terms of hydrophobicity, volume, and the preference for forming α-helices [20]. However, in contrast to Met, Leu is non-oxidizable. In addition, we used methionine sulfoxide reductases (Msr) A and isoform B3A, which reduce stereospecifically (*S*)- and (*R*)-MetSO, respectively [21,22], to determine EF activation by Met-reduced CaM.

**Table 1:** Nomenclature of analyzed CaM-mut with Met (M) to Leu (L) substitutions [19].

CaM	Position in CaM								
	36	51	71	72	76	109	124	144	145
wt	M	M	M	M	M	M	M	M	M
L9	L	L	L	L	L	L	L	L	L
M36/L8	M	L	L	L	L	L	L	L	L
M51/L8	L	M	L	L	L	L	L	L	L
M71/L8	L	L	M	L	L	L	L	L	L
M72/L8	L	L	L	M	L	L	L	L	L
M76/L8	L	L	L	L	M	L	L	L	L
M144/L8	L	L	L	L	L	L	L	M	L
M145/L8	L	L	L	L	L	L	L	L	M
M36,M51/L7	M	M	L	L	L	L	L	L	L
M71,M72,M76/L6	L	L	M	M	M	L	L	L	L
M109,M124/L7	L	L	L	L	L	M	M	L	L
M144,M145/L7	L	L	L	L	L	L	L	M	M
L2	M	M	M	M	M	M	M	L	L

## 2. Results

### 2.1. Ca^2+^-Dependence of CaM Stimulation of EF

**Figure 1:**
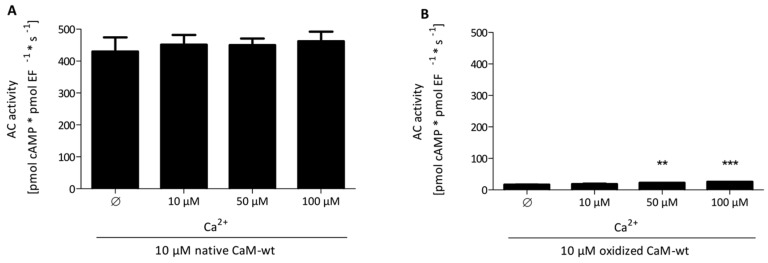
Effect of Ca^2+^-concentration on AC activity of EF stimulated by native or oxidized CaM-wt. The calculation of free Ca^2+^-concentrations, the oxidation of CaM-wt using 50 mM H_2_O_2_ and 0.1 mM CaCl_2_ for 24 h at 25 °C and the AC activity assay were performed as described in the “Experimental Section”. A concentration of 10 μM of native CaM-wt (**A**) or oxidized CaM-wt (**B**) and free Ca^2+^-concentrations of 0 μM, 25 μM, 50 μM, 75 μM, and 100 μM were used in the reaction. The AC activities show the means ± SD of three independent experiments performed in duplicates. A one-way analysis of variances with a Dunnett’s multiple comparison post-test with native or oxidized CaM-stimulated AC activity and 10 μM Ca^2+^ as control was performed to detect significant differences in native or oxidized CaM-stimulated AC activities with various Ca^2+^-concentrations (no *: *p*-value > 0.05; **: 0.001 < *p*-value < 0.01; ***: *p*-value < 0.001). All calculations were performed using GraphPad Prism 5.04.

No remarkable differences were observed in the AC activity of EF stimulated by native CaM-wt in the absence of free Ca^2+^ or in the presence of 10 μM, 50 μM, and 100 μM free Ca^2+^ (Figure 1A). The result of stimulation with completely oxidized CaM-wt was similar, except that the level of basal stimulation was much lower compared to stimulation by native CaM-wt (Figure 1B).

These findings suggest that Ca^2+^-binding to CaM is not necessary for CaM activation of EF although previous studies reviewed in Ref. 6 point to an important role of Ca^2+^ in the regulation of the catalytic activity of EF by CaM. It remains to be determined how Mn^2+^ (used instead of Mg^2+^ in the present study) affects EF regulation by CaM. Based on these results, a concentration of 10 μM free Ca^2+^, analogously to experiments analyzing AC1 activation [19], was used for subsequent studies analyzing CaM stimulation of EF.

### 2.2. Regulation of EF by the Degree of Met Oxidation in CaM

**Figure 2:**
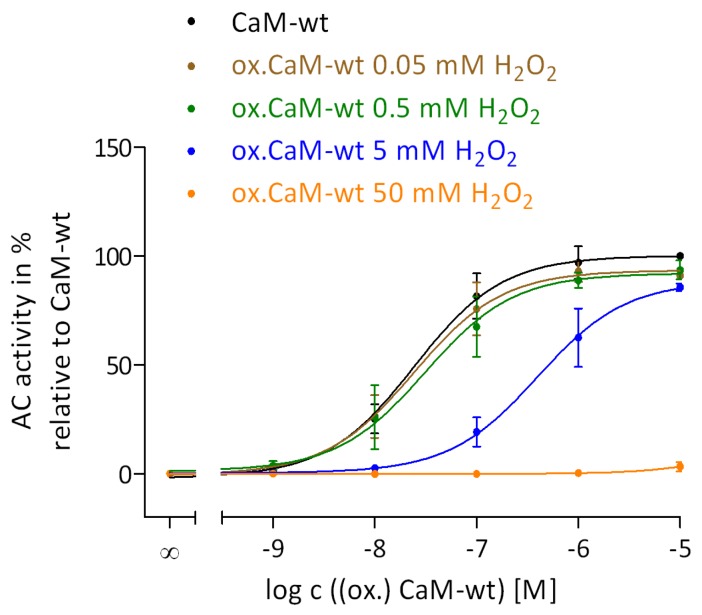
Regulation of EF by the degree of Met oxidation in CaM. Met oxidation using 0.05 mM, 0.5 mM, 5 mM, and 50 mM H_2_O_2_ and 0.1 mM CaCl_2_ for 24 h at 25 °C and the AC activity assay were performed as described in the “Experimental Section”. The concentrations of (oxidized) CaM-wt varied from 1 nM to 10 μM. Concentration-response curves of native CaM-wt (**black**) and oxidized CaM-wt (0.05 mM H_2_O_2_ (**brown**), 0.5 mM H_2_O_2_ (**green**), 5 mM H_2_O_2_ (**blue**), and 50 mM H_2_O_2_ (**orange**)) were analyzed by nonlinear regression (three parameters) using GraphPad Prism 5.04. The AC activity of EF with 30 mM Tris-HCl, pH 7.5 was set to 0% and with 10 μM native CaM-wt to 100%. The AC activities show the means ± SD of three independent experiments performed in duplicates.

**Figure 3:**
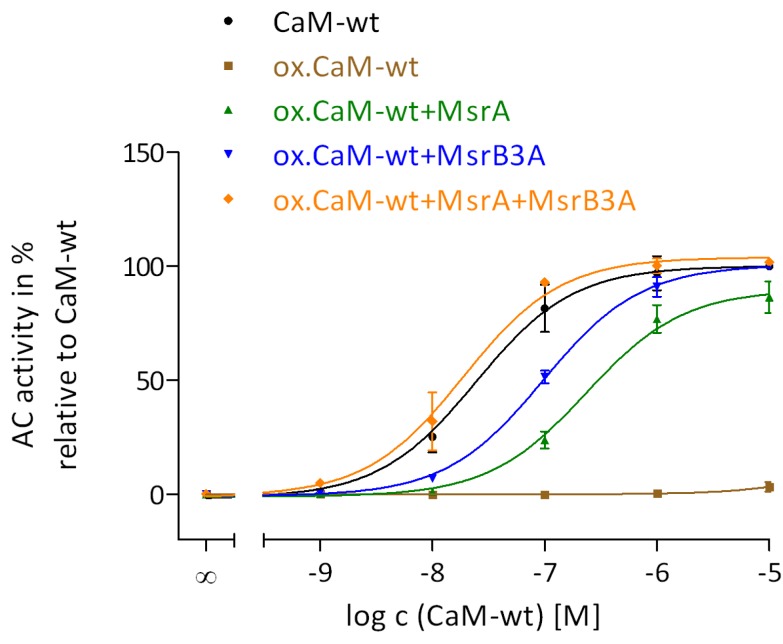
Restoration of CaM activation of EF by reduction of MetSO in oxidized CaM catalyzed by MsrA and MsrB3A. Met oxidation using 50 mM H_2_O_2_ and 0.1 mM CaCl_2_ for 24 h at 25 °C, the treatment of oxidized CaM-wt with MsrA or/and MsrB3A and the AC activity assay were performed as described in the “Experimental Section”. The concentrations of different forms of CaM-wt varied from 1 nM to 10 μM. The concentration-response curves of native (**black**), oxidized (**brown**), MsrA- (**green**), MsrB3A- (**blue**), and MsrA/MsrB3A-treated (**orange**) CaM-wt were analyzed by nonlinear regression (three parameters) using GraphPad Prism 5.04. The AC activity of AC1 with 30 mM Tris-HCl, pH 7.5 was set to 0% and with 10 μM native CaM-wt to 100%. The AC activities show the means ± SD of three independent experiments performed in duplicates.

The effect of Met oxidation in CaM on the ability of CaM to activate EF was performed using samples of CaM oxidized with 0.05 mM, 0.5 mM, 5 mM, or 50 mM H_2_O_2_ (Figure 2). The treatment of CaM with H_2_O_2_ resulted only in Met oxidation whereas no other protein modifications generated were confirmed by the restoration of full EF activation after reduction of MetSO by Msr enzymes (Figure 3).

The concentration-response curves of CaM-wt oxidized with 0.05 mM and 0.5 mM H_2_O_2_ were similar to unoxidized CaM-wt in terms of potency and efficacy. The potency of CaM-wt oxidized with 5 mM H_2_O_2_ was markedly decreased. The decrease in the AC activity of EF in the presence of 10 μM oxidized CaM was marginal relative to the maximal EF activity in the presence of native CaM-wt. No activation of EF was observed by CaM-wt that was heavily oxidized with 50 mM H_2_O_2_ at concentrations of up to 10 μM of oxidized CaM. These results suggest that only the affinity of EF and CaM is affected by the degree of Met oxidation using 5 mM H_2_O_2_, but the maximal CaM stimulation of EF is only affected by completely oxidized CaM with 50 mM H_2_O_2_. In fact, CaM oxidized with 5 mM or 50 mM H_2_O_2_ showed similar degrees of Met oxidation analyzed by SDS-PAGE in a previous study of our group [19], but the above observations confirm the hypothesis that SDS-PAGE is not a suitable method for analyzing subtle differences in the degree of Met oxidation of CaM [19].

### 2.3. Regulation of EF Activation by Native CaM-mut with Met to Leu Substitutions

**Figure 4:**
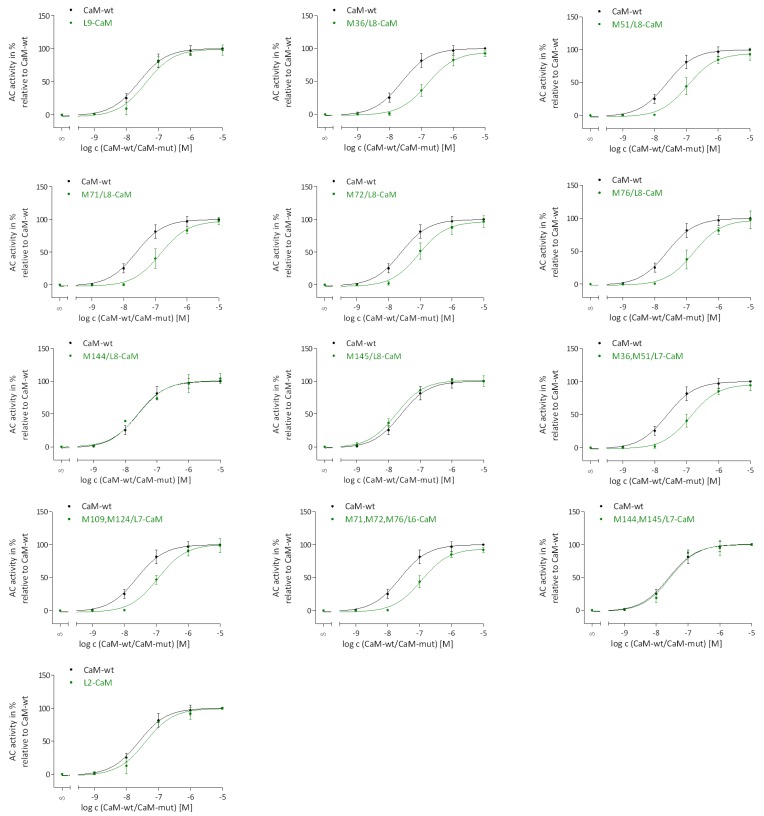
Concentration-response curves for the stimulation of EF by native CaM-wt and CaM-mut. The AC activity assay was performed as described in the “Experimental Section”. Concentrations of CaM-wt (**black**) and CaM-mut (**green**) varied from 1 nM to 10 μM. Concentration-response curves were analyzed by nonlinear regression (three parameters) using GraphPad Prism 5.04. The AC activity of EF with 30 mM Tris-HCl, pH 7.5 was set to 0% and with 10 μM CaM-wt to 100%. The AC activities show the means ± SD of three independent experiments performed in duplicates.

It was necessary first to analyze the impact of Met to Leu substitution on CaM stimulation of EF prior to evaluating the impact of site-specific Met oxidation. Therefore, concentration-response curves of CaM-wt and CaM-mut were recorded (Figure 4).

Site-specific substitutions of Met to Leu in CaM-mut did not alter the maximal AC activity of EF. The potencies of CaM-mut M144/L8, M145/L8 and M144, M145/L7 were not majorly decreased compared to the potency of CaM-wt whereas the potencies of all other CaM-mut were slightly decreased. These observations indicate that the potency is least affected if one Met in position 144 or 145 or two Met in position 144 and 145 are conserved.

### 2.4. Regulation of EF Activation by Site-Specific N- and C-Terminal Met Oxidation

Figure S1 shows that DTT (required for subsequent Msr reactions with oxidized CaM to evaluate the recovery of EF activation by Msr restored oxidized CaM) did not affect the basal AC activity of EF. The results of the recovery of EF activation upon MetSO reduction catalyzed by Msr enzymes are further described in Section 2.5. In order to analyze the impact of site-specific Met oxidation in CaM on EF activation, concentration-response curves shown in Figure S2 were recorded and AC activities, relative to AC activity with each corresponding native CaM-mut, are summarized in Table 2. Corresponding pEC_50_ values are summarized in Table 3.

**Table 2:** Activity of EF stimulated native, oxidized, and MsrA-treated oxidized CaM-wt and CaM-mut. AC activities with 10 μM CaM sample were collected from the corresponding concentration-response curves shown in Figure S2. Met oxidation using 50 mM H_2_O_2_ and 0.1 mM CaCl_2_ for 24 h at 25 °C, the reaction of oxidized CaM-wt and CaM-mut with MsrA and the AC activity assay were performed as described in the “Experimental Section”. Concentration-response curves were analyzed by nonlinear regression (three parameters). The AC activity of EF in the presence of 30 mM Tris-HCl, pH 7.5 was set to 0% and in the presence of 10 μM native CaM-wt or each native CaM-mut was set to 100%. The AC activities show the means ± SD of three independent experiments performed in duplicates. A one-way analysis of variances with a Dunnett’s multiple comparison post-test with native CaM-wt or each corresponding native CaM-mut as control was performed to detect significant differences in AC activities with 10 μM oxidized or with MsrA-treated oxidized CaM-wt or CaM-mut in comparison to native CaM-wt or CaM-mut (**: *p*-value < 0.01; ***: *p*-value < 0.001). All calculations were performed using GraphPad Prism 5.04.

CaM	AC Activity with 10 μM CaM-wt/CaM-mut [%]	
	Oxidized	Oxidized
wt	3.3 ± 2.1 ***	86.3 ± 6.9 ***
L9	100.3 ± 15.9	100.6 ± 5.2
M36/L8	95.7 ± 7.5	97.7 ± 5.0
M51/L8	85.9 ± 15.7	99.1 ± 10.0
M71/L8	91.2 ± 3.0	99.1 ± 4.7
M72/L8	94.9 ± 7.8	101.3 ± 5.2
M76/L8	84.7 ± 12.8	102.7 ± 10.4
M144/L8	101.5 ± 15.4	95.7 ± 2.4
M145/L8	104.9 ± 16.6	100.8 ± 16.7
M36,M51/L7	90.9 ± 9.7	108.4 ± 7.2
M71,M72,M76/L6	86.4 ± 12.5	111.0 ± 7.1
M109,M124/L7	82.4 ± 11.4	106.9 ± 6.9
M144,M145/L7	96.2 ± 6.7	96.3 ± 3.9
L2	74.8 ± 5.8 **	91.3 ± 7.0

**Table 3:** Potencies of EF with native, oxidized, and MsrA-treated oxidized CaM-wt and CaM-mut. pEC_50_ values were calculated from the corresponding concentration-response curves shown in Figure S2 as described in the “Experimental Section”. Concentration-response curves were analyzed by nonlinear regression (three parameters). The pEC_50_ values show the means ± SD of three independent experiments performed in duplicates. A one-way analysis of variances with a Dunnett’s multiple comparison post-test with each corresponding native CaM as control was performed to detect significant differences in potencies of oxidized or MsrA-treated oxidized CaM in comparison to native CaM (**: *p*-value < 0.01; ***: *p*-value < 0.001). All calculations were performed using GraphPad Prism 5.04.

CaM	pEC_50_		
	Native	Oxidized	MsrA-treated
wt	7.61 ± 0.05	-	6.35 ± 0.09 ***
L9	7.40 ± 0.10	7.31 ± 0.13 **	6.96 ± 0.05 ***
M36/L8	6.81 ± 0.06	6.50 ± 0.06 ***	6.29 ± 0.04 ***
M51/L8	6.96 ± 0.09	6.83 ± 0.14 **	6.50 ± 0.07 ***
M71/L8	6.83 ± 0.08	6.35 ± 0.05 ***	6.35 ± 0.05 ***
M72/L8	7.05 ± 0.07	6.75 ± 0.07 ***	6.57 ± 0.05 ***
M76/L8	6.81 ± 0.10	6.47 ± 0.10 ***	6.37 ± 0.06 ***
M144/L8	7.54 ± 0.11	7.08 ± 0.10 ***	6.86 ± 0.04 ***
M145/L8	7.73 ± 0.05	7.14 ± 0.10 ***	6.97 ± 0.07 ***
M36,M51/L7	6.88 ± 0.07	6.19 ± 0.06 ***	6.29 ± 0.04 ***
M71,M72,M76/L6	6.94 ± 0.06	6.30 ± 0.07 ***	6.27 ± 0.04 ***
M109,M124/L7	6.95 ± 0.05	5.90 ± 0.06 ***	6.35 ± 0.04 ***
M144,M145/L7	7.56 ± 0.09	6.52 ± 0.06 ***	6.92 ± 0.05 ***
L2	7.40 ± 0.10	5.33 ± 0.07 ***	6.62 ± 0.05 ***

In addition to the complete loss of EF activation upon stimulation with oxidized CaM-wt, a significant decrease (25%) in EF activation was evident for oxidized L2-CaM. The remaining results show that maximal EF activation was not affected by oxidation of one, two, or three Met in other CaM-mut. The potencies were substantially decreased for all oxidized CaM-mut, except for L9-CaM, demonstrating only a nominal decrease in potency. This indicates that Met oxidation has a remarkable impact for the affinity of CaM to EF, but not for the maximal stimulation.

### 2.5. Restoration of CaM Stimulation of EF by Msr Catalyzed Reduction of MetSO

To investigate the recovery of CaM stimulation of EF by restored oxidized CaM, MsrA and MsrB3A were used to reduce (*S*)-and (*R*)-MetSO, respectively. The restoration of (*R*)-MetSO by MsrB3A was performed exemplarily using oxidized CaM-wt, but not all CaM-mut, because the restoration in detail was not the aim of this study. Figure 4 shows that maximal CaM activation of EF was almost completely restored by MsrA or MsrB3A catalyzed reduction of MetSO in oxidized CaM.

The potency of oxidized CaM-wt that was treated with either of the Msr enzymes, was decreased in comparison to native CaM-wt. In contrast, full EF activation and potency levels of native CaM-wt were obtained upon treatment of oxidized CaM-wt with both Msr enzymes. Complete recovery of EF activation was also observed for oxidized L2-CaM upon MsrA treatment (Table 2). However, decreased potencies of oxidized CaM-mut were not regained by MsrA restoration possibly due to structural changes accompanying Met oxidation that were not restored by MetSO reduction. In L9-CaM, all Met that were prone to oxidation were substituted with Leu and, as expected, this CaM-mut was resistant to oxidation and unaffected by MsrA treatment.

## 3. Discussion

### 3.1. Impact of Intact C-Terminal Structure of CaM on EF Activation

Regulation of EF by oxidized CaM has pathophysiological relevance because ROS, generated by the NADPH-oxidase in activated neutrophils [11], can oxidize susceptible amino acid residues in host proteins like CaM [13,14]. EF was fully activated by CaM-wt oxidized with 5 mM H_2_O_2_ with only a moderate decrease in potency. However, with completely oxidized CaM-wt (50 mM H_2_O_2_) with all nine Met residues oxidized to MetSO (as shown by previous mass spectrometry analysis [19,23]), no EF activation was observed (Table 2). In contrast, EF activation was not affected by oxidized CaM-mut, which contains up to three MetSO (Table 2). Furthermore, the oxidation of seven Met in L2-CaM resulted only in a modest decrease of EF activation of about 25%. Evidently, for any particular Met in CaM, the polarity and rigidity differences accompanying oxidation to MetSO [20,24] do not affect the ability to activate EF. Only the apparent affinity between EF and CaM is decreased by oxidation (Table 3) supporting the concept that binding of CaM to EF and EF activation are tightly coupled, and there is little nonproductive binding [6,10]. Thus, binding of CaM to EF is impaired by CaM Met oxidation, but if the CaM-EF complex is already built, full EF activation is possible even though Met in CaM are oxidized. The data also indicate that binding of CaM to EF is affected by the secondary structure of CaM, which is suggested to be associated with Met oxidation. We have shown that the α-helical content of CaM-wt and L2-CaM decreases only moderately, but significantly, upon oxidation [19]. It is reasonable that the reduction of the α-helical content, especially in the N-terminal region due to oxidation of the *N*-terminal Met residues (M36, M51, M71, M72 and M76), is critical for binding of CaM to EF. Our data confirm that the N-terminal region of CaM is important for the initiation of CaM-EF binding [6,25].

**Figure 5:**
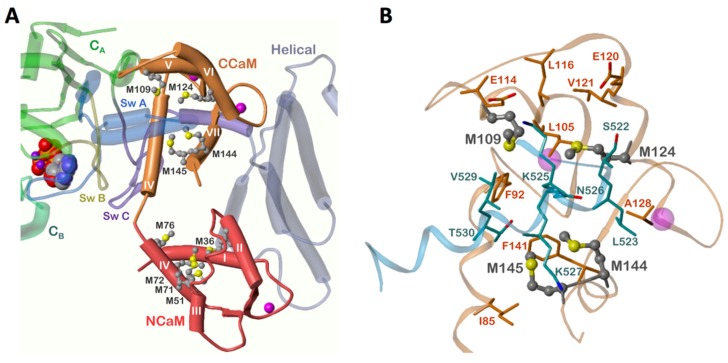
Positions of Met residues in CaM within the CaM-EF complex. The models were generated with Sybyl 8.0 (Certara, St. Louis, MO, USA) using the crystal structure PDB 1xfv [26]. Met residues are drawn as ball and stick models. Atom colors: C, grey; O, red; N, blue; P, orange; S, yellow; Mg^2+^, purple; Ca^2+^, magenta. CaM, calmodulin; Met, methionine; Sw, switch. (**A**) Overview of the CaM structure and surrounding EF domains. Helices are presented as cylinders, β-sheets as arrows, loops and turns as tubes, 3′-deoxy-ATP as space fill model. (**B**) Interaction of C-terminal M109, M124, M144, and M145 with other amino acids of C-CaM and switch A of EF. All residues within a sphere of 3 Å around at least one Met are shown. C-CaM, orange; switch A of EF, blue.

A remarkable finding of this study is that EF was still activated to 75% by oxidized L2-CaM (Leu residues at positions 144 and 145), compared to the complete loss of EF activation with oxidized CaM-wt, at the highest concentrations of CaM used. Furthermore, the data for L2-CaM (Figure S2) suggest that higher concentrations of L2-CaM would fully activate EF, again suggesting a lack of nonproductive binding. From the crystal structure of CaM and EF it is known that M109, M124, M144, and M145 are potentially important for EF activation because of the large surface area they contribute to the CaM-EF interface [10]. These Met residues are in mainly hydrophobic contact to amino acids in switch A of EF within the CaM-EF complex that is in contrast to the *N*-terminal Met residues (this is demonstrated via molecular modeling illustrated in Figure 5).

The above data indicate that an intact structure in the *C*-terminal region of oxidized CaM, especially next to positions 144 and 145, is sufficient for effective EF activation. This is evident despite the structural changes associated with Met oxidation in the *N*-terminal region [6,25]. Furthermore, full EF activation was restored by treatment of oxidized CaM-wt and L2-CaM with either MsrA or MsrB (Table 2 and Figure 3), suggesting that partial restoration of CaM-structure by only one Msr enzyme is sufficient for EF activation [18,23]. Upon restoration of oxidized CaM-wt by both Msr enzymes full CaM potency was regained (Figure 3).

The decrease in Ca^2+^-affinity of CaM upon Met oxidation has to be considered as well [24]. Full EF activation was obtained with oxidized CaM-mut M109,M124/L7 and M144,M145/L7, where two oxidized Met residues are located in vicinity to the *C*-terminal Ca^2+^-binding sites, respectively [27]. These findings reveal that Ca^2+^-binding in the *C*-terminal region of CaM (important for EF activation (in contrast to Ca^2+^-binding in the *N*-terminal region) [10,28]) is sufficient for EF activation upon oxidation of either M109 and M124 or M144 and M145. Moreover, effective EF activation generally seems to be not necessarily dependent on Ca^2+^-binding to CaM (Figure 1), initiation of binding of CaM and EF [6,25] is ensured by an intact N-terminal structure of these CaM-mut and a partially intact structure of the *C*-terminal region. The importance of M144 and M145 for EF activation is further shown by analysis of native CaM-mut with Met to Leu substitutions because only if M144 and M145 (in CaM-mut M144/L8, M145/L8 and M144,M145/L7) are not substituted to Leu, the affinity to EF is almost identical to the affinity of CaM-wt to EF (Figure 4).

### 3.2. Comparison of CaM-EF Interaction to Other CaM-Target Interactions

The results for CaM activation of EF observed in this study are consistent with previous findings demonstrating that CaM regulation of EF is very different from CaM regulation of a mammalian membranous AC1 [6,10]. Site-specific Leu substitutions in CaM-mut enhanced AC1 activation dramatically in comparison with CaM-wt [19]. In contrast, EF activation is not affected by Met to Leu substitutions (Figure 3). Only the affinity of EF and CaM was altered accompanying particular Met to Leu substitutions (Figure 3). AC1 was also more sensitive to site-specific Met oxidation than EF. It is worth mentioning that AC1 activation by CaM is strongly Ca^2+^-dependent, whereas EF is also activated by apo-CaM (Figure 1 and [19]), indicating that the CaM binding surface of EF is more adaptable to different tertiary structures of CaM. Furthermore, CaM activates AC1 with relatively low affinity, whereas CaM activates EF with relatively high affinity [19].

Similar results to the current EF activation by oxidized CaM and Msr-treated CaM were obtained for CyaA, another CaM-stimulated AC toxin. CyaA is the AC toxin of *Bordetella pertussis*, the causative agent of whooping cough [18]. EF and CyaA exhibit similar catalytic and regulatory mechanisms, but their CaM-binding sites possess different characteristics [6,10,17]. For CyaA activation, M109, M124, and M145 were identified as the most important Met residues in CaM [18]. In the current study, especially Met at position 144 and 145, were identified as important for effective EF activation. In accordance with previous findings [6,25], our data suggest that N-terminal Met residues in CaM are critical for binding to EF (Table 2 and Figure 5). In contrast, CyaA activation is possible with only the *C*-terminal region of CaM [25]. A comparison of particular Met residues important for CaM activation of three CaM-stimulated ACs (membranous AC1, *Bacillus anthracis* AC toxin EF and *Bordetella pertussis* AC toxin CyaA) is summarized in Table 4.

Hydrophobicity, flexibility, and size of amino acids in distinct positions play important roles in AC1 activation, as shown by the increased activation of AC1 by several CaM-mut [19], in comparison to minimal or no differences for activation of EF, CyaA, plasma membrane Ca^2+^-ATPase (PMCA), neuronal or endothelial nitric oxide synthase (NOS), and cyclic nucleotide phosphodiesterase (PDE) based on Met to Leu substitutions in positions 144 or 145 [29,30,31,32].

For most CaM-targets, including EF, that have been analyzed so far, Met residues in the *C*-terminal region are essential for activation [16,18,31,32]. For instance, oxidation of M144 or M145 impairs activation of EF, PMCA, and neuronal NOS. The oxidation of M109, M124, and M145 and Met to Leu substitution of M145 impair CyaA activation, suggesting that M109, M124, and M145 are important for CaM-CyaA interaction, whereas *N*-terminal Met residues and M144 are less relevant [18,29]. Concerning the simultaneous oxidation of M144 and M145, the findings are not consistent among all targets: CaM with two MetSO in positions 144 and 145 did not fully activate PMCA [31], but did fully activate EF (Table 2), AC1 [19], and neuronal and endothelial NOS [32]. Structural changes in the position of the linker and the globular domains resulting from oxidation of M144 and M145 [33] apparently have different importance for individual CaM-targets, but not for all. For activation of PMCA, structural changes following Met oxidation are more important than the changes of the polarity by MetSO per se [34]. In contrast, for the CaM-AC1 interaction, changes in the flexibility and the polarity of distinct amino acids are also of great importance, in addition to perturbations of the CaM structure.

**Table 4:** Relevance of specific Met residues in CaM for regulation of membranous AC1, *Bacillus anthracis* AC toxin edema factor and *Bordetella pertussis* AC toxin CyaA by oxidation to MetSO.

M ^a^	Membranous AC1 ^b^	*Bacillus anthracis* AC toxin EF	*Bordetella pertussis* AC Toxin CyaA ^c^
36	High relevance: Single Met in the *N*-terminal region of CaM enhances AC1 activation and MetSO impairs AC1 activation	Moderate relevance: Important for CaM-EF binding, oxidation decreases CaM-EF affinity; structure: No contact to CaM within the CaM-EF complex	No relevance: Met is accessible to oxidation within the CaM-CyaA complex; oxidation does not prevent binding to CyaA
51	High relevance: MetSO impairs AC1 activation	Moderate relevance: Like M36	No relevance: Like M36
71	Only relevant in combination of oxidized M72, M72 and M76: AC1 activity decreases significantly	Moderate relevance: Like M36	No relevance: Like M36
72	Like M71	Moderate relevance: Like M36	No relevance: Like M36
76	Like M71	Moderate relevance: Like M36	No relevance: Like M36
109	High relevance in combination of oxidized M109 and M124: AC1 activity decreases significantly	Low relevance: Oxidation of M109 and M124 does not affect EF activation; structure: Met is in contact with CaM within the CaM-EF complex	High relevance: Crystallographic studies reveal important hydrophobic interactions with CyaA; preservation of this Met from oxidation within the CaM-CyaA complex and prevention of binding to CyaA in oxidized state suggest an involvement in the formation of CaM-CyaA complex
124	High relevance: Like M109	Low relevance: Like M109	High relevance: Like M109
144	High relevance: Met enhances AC1 activation and MetSO impairs AC1 activation; no altered AC1 activation in combination of two Met or MetSO at position 144 and 145	High relevance: An intact *C*-terminal region next to M144 and M145 is sufficient for effective EF activation also upon oxidation of all other Met, important for the high affinity of EF to CaM; molecular modeling: Met is in contact with CaM within the CaM-EF complex	Moderate relevance: Crystallographic studies reveal important hydrophobic interactions with CyaA; Met is accessible to oxidation within the CaM-CyaA complex; site-specific oxidation does not prevent binding to CyaA but in combination with other C-terminal Met oxidation prevents binding to CyaA
145	High relevance: Like M144	High relevance: Like M144	High relevance: Like M109, additionally: Important for CyaA activation

^a^ M, methionine; ^b^ Based on experimental data shown in [19]; ^c^ Based on experimental data shown in [17,18,29].

The sensitivity to oxidative modifications in CaM differs also between CaM-targets. An about half-maximal stimulation of PMCA and CyaA was preserved with oxidized CaM-wt compared to unoxidized CaM-wt [18,31]. A complete loss of function of oxidized CaM-wt oxidized was observed for AC1 and EF activation (Table 2 and [19]), revealing a higher sensitivity of these targets to oxidative modifications in CaM. However, CaM-wt that is not completely oxidized (oxidized with only 5 mM H_2_O_2_) led to a major loss of AC1 activation, but not of EF activation (Figure 2 and [19]), indicating that AC1 is the most sensitive enzyme among CaM-targets compared. A similar pattern was detected after reduction/repair of oxidized CaM-wt catalyzed by MsrA or/and MsrB. Partial repair of distinct Met residues by MsrA or MsrB, and the associated restoration of native-like structure of CaM-wt, was sufficient for full activation of EF (Figure 4), CyaA [18], and PMCA [23], whereas full activation of AC1 was only restored when oxidized CaM-wt was treated with both Msr enzymes in combination [19]. Hence, restoration of all nine MetSO is important for full AC1 activation but not for full EF, CyaA, or PMCA activation, where restoration of the native-like CaM structure by MetSO reduction is apparently more relevant. [18,19,23].

To summarize, the contribution of individual Met residues in CaM to activation of AC1 is more intricate than activation of both AC toxins EF and CyaA. The toxins are well adapted to oxidative stress conditions (occurring in activated neutrophils during inflammation) and to tertiary structure perturbations of CaM accompanying Met oxidation. These differences in CaM stimulation of the three ACs suggest good prospects for developing new inhibitors of EF and CyaA, targeting their interactions with CaM, that would selectively inhibit the toxins, but not mammalian AC1 [6,9].

## 4. Experimental Section

### 4.1. Materials

Bovine serum albumin was purchased from Sigma-Aldrich (Taufkirchen, Germany). Bio-Rad DC protein assay kit was obtained from Bio-Rad (Hercules, CA, USA). [α-^32^P]ATP (3000 Ci/mmoL) was purchased from Hartmann (Braunschweig, Germany). Sources of other materials for EF expression and purification, AC activity assay and CaM oxidation and reduction were obtained as previously described [19,35].

### 4.2. Expression and Purification of EF

The plasmid pProExH6-EF was prepared and amplified in *Escherichia coli* BL 21(DE3)/pUBS520 cells as previously described [10,25,26,36]. Expression and purification of EF were performed as previously described [25] with minor modifications [35].

### 4.3. Cloning, Expression, and Purification of CaM-wt and CaM-mut

Mutagenesis, expression in *Escherichia coli* and purification of CaM-wt and all CaM-mut were performed as previously described [19,31] and based on the coding region for chicken CaM (Uni-Prot entry P62149). This gene code is identical to human CaM (Uni-Prot entry P62158). The numbering of the amino acids used in this study (A1-K148) neglects an *N*-terminal Met because it is cleaved during expression. The CaM (in 1 mM imidazole, pH 6.5, 0.1 mM KCl and 10 mM CaCl_2_) was exhaustively dialyzed against deionized water before its usage to remove all buffer components and Ca^2+^. CaM concentration was determined by the Lowry method [37] using the Bio-Rad DC protein assay kit. Therefore, bovine serum albumin serves as the standard. The nomenclature of analyzed CaM-mut is shown in Table 1.

### 4.4. Oxidation of Met and Reduction of MetSO

Met oxidation was performed as previously described [19]. Briefly, 60 μM CaM dissolved in deionized water was incubated for 24 h at 25 °C with 0.1 mM CaCl_2_ and H_2_O_2_ at various concentrations. After the oxidation step, Ca^2+^ and H_2_O_2_ were exchanged against deionized water for AC assays or against 30 mM Tris-HCl, pH 7.5 for the treatment with the Msr enzymes.

Expression and purification of Msr enzymes and reduction of MetSO were performed as previously described [19,38,39,40]. For the reduction of oxidized Met, 60 μM oxidized CaM was incubated either with 4 μM MsrA or MsrB3A or with 2 μM of each MsrA and MsrB3A in the presence of 10 mM DTT for 1 h at 37 °C. Msr enzymes or DTT did not affect the AC activity of EF (Figure S1).

### 4.5. AC Activity Assay

AC activity of EF was determined as previously described [35] with some modifications. Briefly, EF (10 pM, dissolved in 20 μL of 45 mM HEPES (2-[4-(2-hydroxyethyl)piperazin-1-yl]ethanesulfonic acid), pH 7.4, containing bovine serum albumin (0.1% (*m*/*v*))), was pre-incubated for 2 min at 25 °C with 10 μL of 30 mM Tris-HCl, pH 7.5 in the presence or absence of native, oxidized, or oxidized CaM-wt/CaM-mut that were treated with MsrA and/or MsrB3A. The concentration of CaM samples varied from 1 nM to 10 μM. Reactions were initiated by adding 20 μL of a reaction mixture consisting of 100 μM cAMP, 40 μM ATP, 100 mM KCl, 5 mM MnCl_2_, 100 μM EGTA and 0.2 μCi [α-^32^P]ATP. The concentration of CaCl_2_ was generally chosen to achieve a free Ca^2+^-concentration of 10 μM except for the data determined in Figure 1 in the presence of 25 μM, 50 μM, 75 μM, and 100 μM free Ca^2+^ and in the absence of free Ca^2+^. EGTA was added to keep the free Ca^2+^ concentration constant. WebMax C standard (http://www.stanford.edu/~cpatton/webmaxcE.htm) was used to calculate free Ca^2+^- and Mn^2+^-concentrations under consideration of buffer components, free metal concentration, pH, and temperature. Reactions were conducted for 10 min at 25 °C and were stopped by adding 20 μL HCl. In order to separate the radioactive labeled product from the educt, samples were transferred onto columns filled with 1.3 g of aluminium oxide (MP Alumina N Super I). [^32^P]cAMP was eluted from columns with 4 mL of 0.1 M ammonium acetate, pH 7.0 and its concentration was measured by Čerenkov radiation.

### 4.6. Statistics

Concentration-response curves of AC activity were analyzed by non-linear regression (three parameters) using GraphPad Prism 5.04 (GraphPad Software Inc., La Jolla, CA, USA). The AC activities with 10 μM (oxidized) CaM-wt/CaM-mut were compared with ANOVA with Dunnett’s multiple comparison post-test.

## 5. Conclusions

In conclusion, our results suggest that bacterial EF activation is highly resistant to oxidative modifications in CaM. This is necessary in order for the bacteria to evade host defenses, in this case the oxidative conditions in activated neutrophils. The unusually large binding region between CaM and EF [10] contributes to the compensatory mechanism for activation by oxidative damaged CaM. Furthermore, our results suggest that an intact structure of the C-terminal region of oxidized CaM is important for EF activation. Interestingly, M144 and M145 are rarely sensitive to oxidation *in vivo* [33] supporting the idea that EF activation is evolutionarily well adapted to oxidative conditions.

## Supplementary Materials

Supplementary materials can be accessed at: http://www.mdpi.com/2072-6651/7/7/2598/s1.
