# Supplementary material for: Different Roles of N-Terminal and C-Terminal Domains in Calmodulin for Activation of Bacillus anthracis Edema Factor
Source: Toxins (Basel). 2015 Jul 13;7(7):2598–614. doi: 10.3390/toxins7072598 (PMC4516931; doi:10.3390/toxins7072598)
Supplement: Supplementary file 1 [file toxins-07-02598-s001.pdf]

Supplementary Information

Different roles of *N*-terminal and C-terminal domains in calmodulin for activation of *Bacillus anthracis* edema factor.

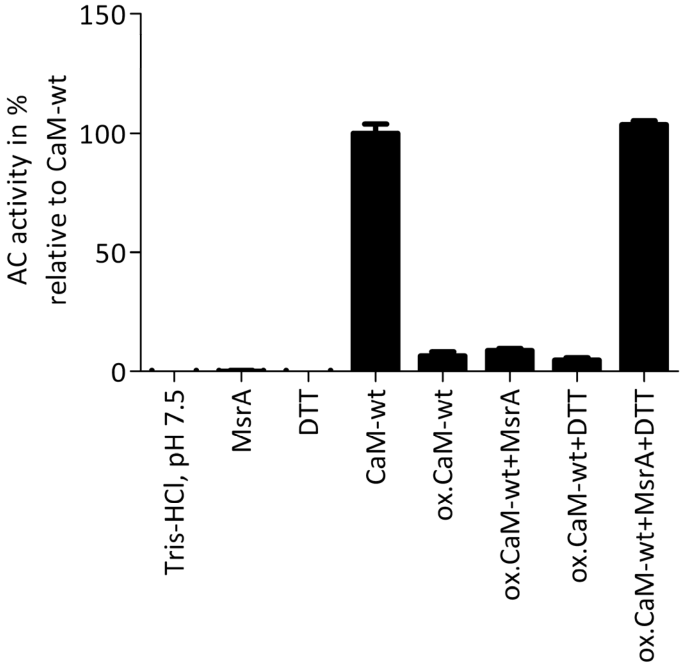

Figure S1. Restoration of AC activity of EF by MsrA-treated oxidized CaM-wt. Reactions contained a final concentration of 10 µM native CaM-wt or oxidized CaM-wt, 0.67 µM MsrA and 2 mM DTT. Treatment of oxidized CaM-wt with 10 mM DTT and/or 4 µM MsrA for 1 h at 37 °C was performed as described in the "Experimental Section". The AC activities show the means ± SD of one experiment performed in triplicates.

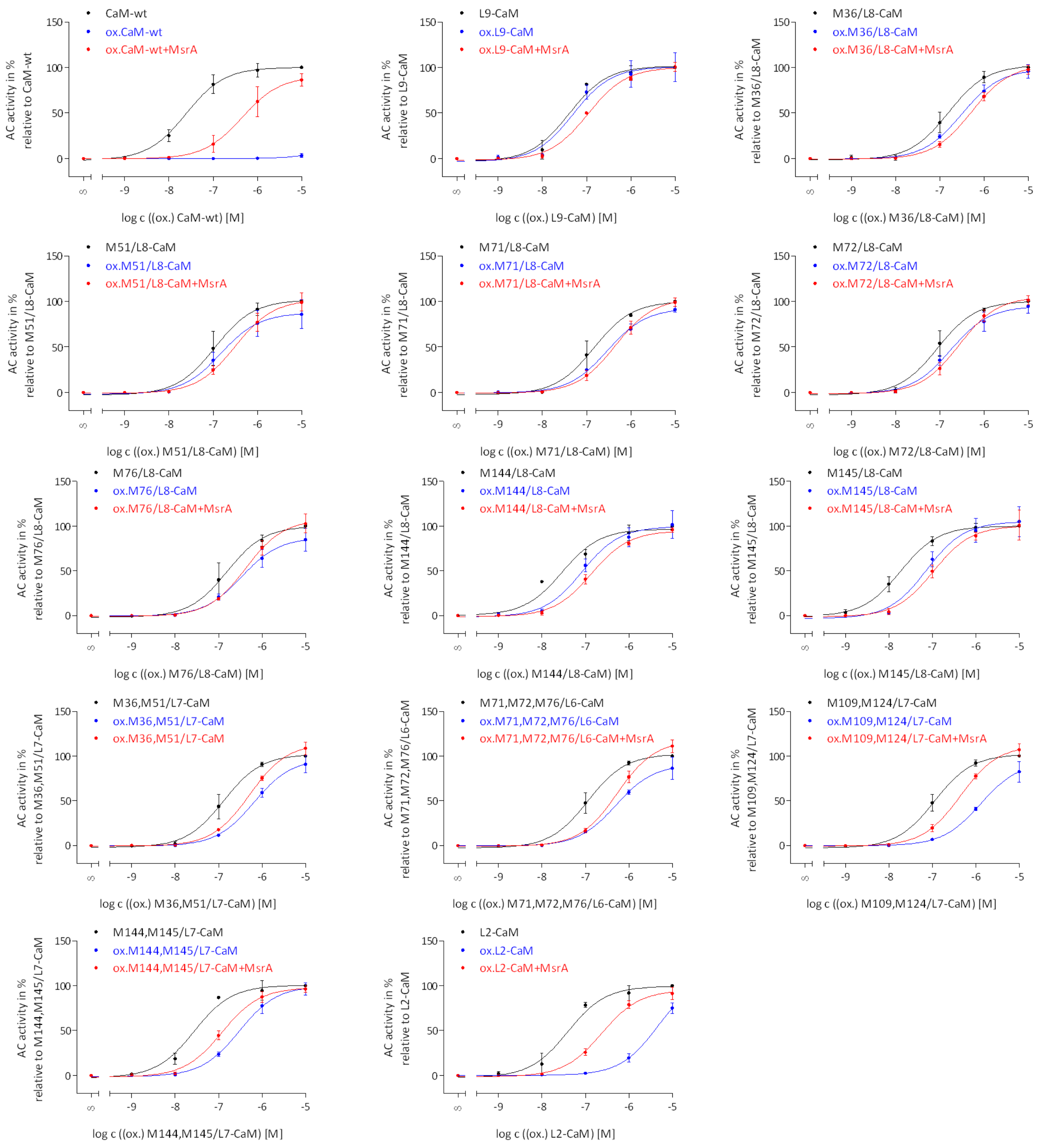

Figure S2. Concentration-response curves for the stimulation of EF by native, oxidized and MsrA-treated oxidized CaM-wt and CaM-mut. Met oxidation using 50 mM H_2O_2 and 0.1 mM $CaCl_2$ for 24 h at 25 °C, the treatment of oxidized CaM-wt or CaM-mut with MsrA and the AC activity assay were performed as described in the "Experimental Section". Concentrations of native (**black**), oxidized (**blue**) and MsrA-treated oxidized (**red**) CaM-wt/CaM-mut varied from 1 nM to 10 µM. Concentration-response curves were analyzed by nonlinear regression (three parameters) using GraphPad Prism 5.04. The AC activity of EF with 30 mM Tris-HCl, pH 7.5 was set to 0% and with 10 µM native CaM-wt or each native CaM-mut to 100%. The AC activites show the means ± SD of three independent experiments performed in duplicates.
